# Hybrid ameloblastoma and central giant cell lesion: Challenge of early diagnosis

**DOI:** 10.4317/jced.56441

**Published:** 2020-02-01

**Authors:** Rúbia-Teodoro Stuepp, Luiz-Henrique-Godoi Marola, Filipe Modolo, Rogério Gondak

**Affiliations:** 1 Postgraduate Program in Dentistry, Federal University of Santa Catarina, Florianopolis, Santa Catarina, Brazil; 2 Bucomaxillofacial Residence Program, University Hospital, Federal University of Santa Catarina, Florianopolis, Santa Catarina, Brazil; 3 Department of Pathology, Federal University of Santa Catarina, Florianopolis, Santa Catarina, Brazil

## Abstract

Hybrid lesions encompass the occurrence of different entities in one lesion. A 67-year-old woman was referred to the Oral and Maxillofacial Surgery Service for treatment of mandibular Central Giant Cell Lesion (CGCL) previously diagnosed. Intraoral examination revealed edentulism and a painless swelling extending from the alveolar ridge to the buccal vestibule with hard consistency covered by normal mucosae, with unknown duration. Panoramic radiograph revealed a large, multilocular and well-defined radiolucent lesion extending from the region of left mandibular lateral incisor teeth to right mandibular first molar with no evidence of osseous perforation. Initially, a treatment with intralesional injection of corticosteroids was performed. After 18 months of treatment, an increase in size of the osteolytic lesion was noted. An incisional biopsy was carried out and the microscopic examination revealed a unicystic ameloblastoma associated to CGCL. It was performed marsupialization and later the enucleation of residual lesion. The follow-up remains being performed.

** Key words:**Hybrid lesion, central giant cell lesion, ameloblastoma.

## Introduction

Hybrid lesions encompass elements of different pathologies in one lesion and the occurrence within the jaws are rarely reported (1). Among these, the mostly reported hybrid lesions are odontogenic lesions: ameloblastoma and glandular odontogenic cyst, ameloblastoma and orthokeratinised odontogenic cyst, ameloblastoma and odontogenic keratocyst, and unicystic ameloblastoma with odontogenic keratocyst (2).

Central giant cell lesion (CGCL) is a benign lesion of the jaws with an unknown etiology. These lesions occur more frequently in females until third decade of life and are often located on the anterior region of mandible. Most cases presents as a intraoral slow growing and painless swelling. The radiological features are diverse and may vary from small unilocular to extensive multilocular radiolucent areas, beside displacement of teeth and tooth germs, root resorption, and cortical perforation. Histopathologically, CGCL is characterized by a cellular connective tissue permeated by mesenchymal ovoid and multinucleated giant cells of various sizes and, occasionally, with multiple foci of hemorrhage and trabecular bone tissue (3).

Chuong *et al.* classified aggressive and non-aggressive lesions according to signs and symptoms and histological features. The lesion is considered aggressive when several of the following features are present such as pain, paresthesia, root resorption, rapid growth, cortical perforation, and high recurrence rate. On the other hand, the non-aggressive lesion is characterized by slower growth and absence of cortical perforation or tooth resorption (4). With regard to the histopathological features, aggressive CGCL has a larger fractional area occupied by giant cells (3). This classification determine the treatment, that can be either conservative with intralesional corticotherapy followed by enucleation either more radical, with surgical resection (3).

Ameloblastoma is as a benign odontogenic tumor with origin in odontogenic epithelial cells (5). Unicystic Ameloblastoma (UA) is a variant form that mostly occurs in a younger age group having an odontogenic cyst-like behavior. UA frequently appears as a unilocular radiolucent with a history of slow growing in mandible (5,6). Histologically, it is characterized as a cystic lesion lined by an ameloblastomatous epithelial lining with or without luminal and/ or mural tumor growth (6), which determine its subclassification. Because of the relatively benign biologic behavior, UA has good response to conservative treatment. Then, enucleation with or without a previous marsupialization is indicated with a low rate of recurrence (5,6).

Both UA and CGCL are uncommon diseases and their occurrence simultaneously with another lesion is very rare. Here we present a case of simultaneous occurrence of CGCL and UA. To the best of our knowledge, this is the first reported case of a simultaneous occurrence of these two entities.

## Case Report

A 67-year-old woman was referred to the service of Oral and Maxillofacial Surgery for surgical treatment of mandibular CGCL previously diagnosed. No alterations were observed on extraoral examination. Intraoral examination revealed a slowly and painless expansive lesion in the left parasymphysis of an unknown duration. The patient was edentulous and the swelling extended from anterior mandibular region to right posterior mandibular region, the mandibular vestibule had a hard consistency, and was covered by normal mucosae.

**Figure 1:**
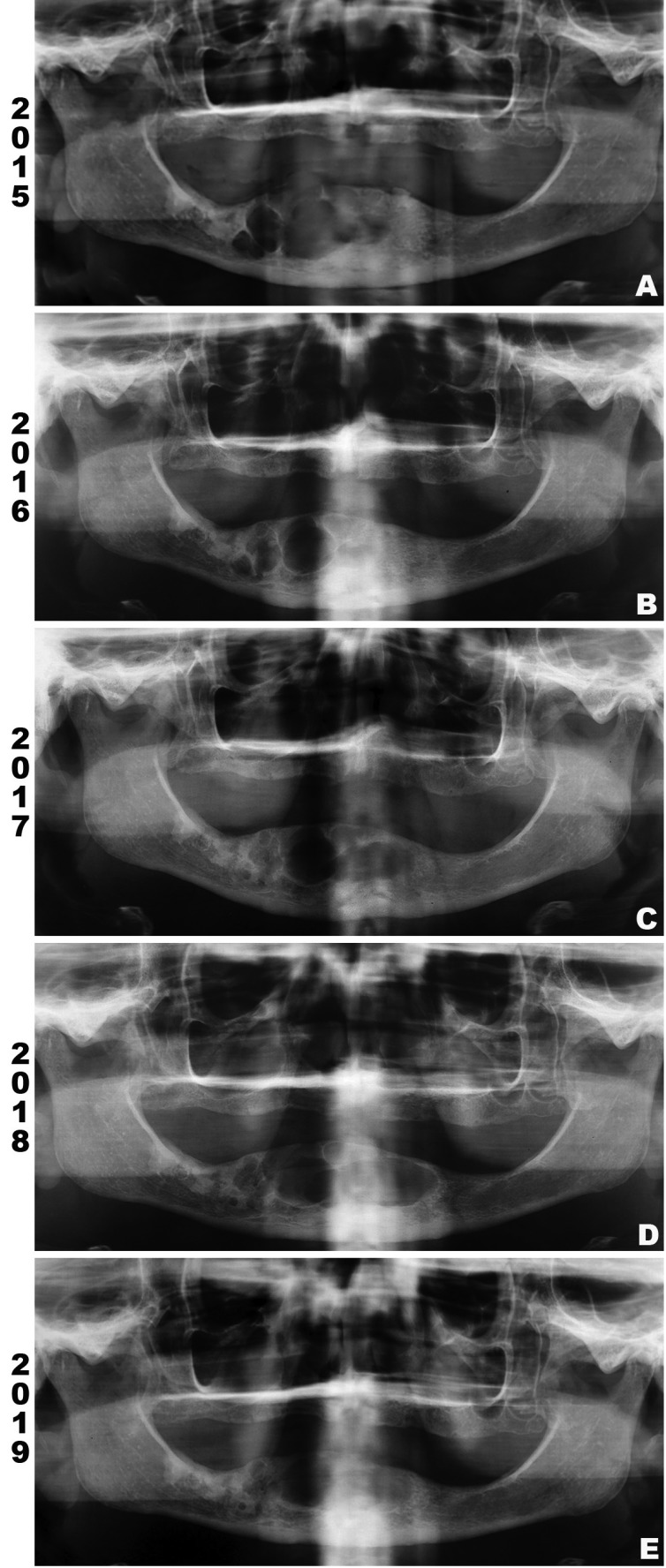
Panoramic radiograph (PR). (A) PR taken in 2015 showing a large, multilocular, well-defined radiolucent lesion extending from the region of left mandibular lateral incisor to right mandibular first molar teeth. (B) PR taken in 2016 indicating decreases of the lesion on right posterior region of mandible. (C and D) PR from 2017 and 2018, respectively, revealing increase of osteolytic component on anterior region of mandible, extending to the left side. (E) PR from 2019 shows increased radiopacity of the lesion, indicating bone repair.

Medical history of the patient revealed systemic arterial hypertension controlled by oral drugs. A panoramic radiograph from 2015 revealed a large, multilocular, well-defined radiolucent lesion extending from the region of left mandibular lateral incisor to right mandibular first molar teeth, with no evidence of cortical perforation (Fig. 1a). At this moment, it was proposed a conservative treatment with intralesional injection of corticosteroids to decrease the size of the lesion.

During 18 months, the patient received monthly 1 ml of Dexamethasone (4mg/ml) applied intralesionally. To reassess the response to the treatment, panoramic radiographs were performed in Dec/2016 (Fig. 1b), Aug/2017 (Fig. 1c) and Feb/2018 (Fig. 1d). In the first radiography, the lesion decreased, specially in the right posterior region of mandible. However, the two last exams revealed an increase of the osteolytic component of the lesion in the anterior region of mandible, extending to the left side and causing an expansion of cortical bone on intraoral clinical examination.

**Figure 2:**
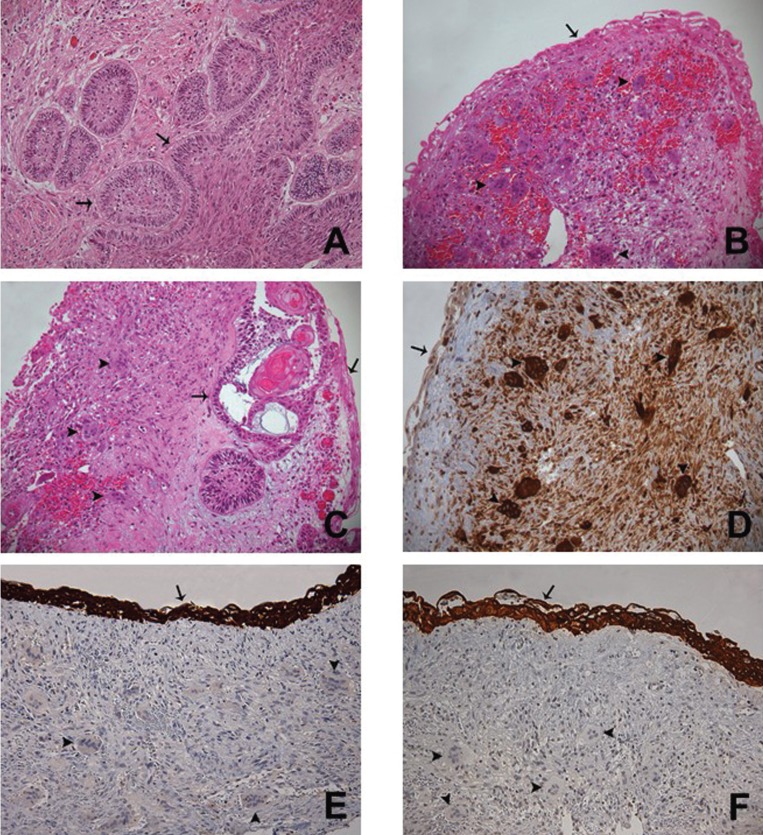
Photomicrographs of histological section on 200x. (A) Histological section (H&E) of a mural proliferation area of UA showing ameloblastomatous epithelium (arrow) exhibiting palisaded basal cells with hyperchromatic nuclei, focal reverse polarization and upper layers with stellate-reticulum-like cells. (B) Ameloblastomatous epithelium (arrow) lining a connective tissue permeated by multinucleated giant cells (arrowhead) of different sizes and ovoid mesenchymal cells (H&E). (C) Connective tissue wall partially lined by ameloblastomatous epithelium (arrow), presenting mural invasion (arrow) and focal areas of multinucleated giant cells (arrowhead) of different sizes (H&E). (D) Connective tissue lined by ameloblastomatous epithelium (arrow) and permeated by multinucleated giant cells (arrowhead) and ovoid mesenchymal cells showing immunoreactivity for CD68. (E) Ameloblastomatous epithelium (arrow) showing immunoreactivity for CK19 and lining a cellular fibroblastic connective tissue permeated by multinucleated giant cells (arrowhead) and ovoid mesenchymal cells. (F) Ameloblastomatous epithelium (arrow) showing immunoreactivity for pan-CK and lining a cellular fibroblastic connective tissue permeated by multinucleated giant cells (arrowhead) and ovoid mesenchymal cells.

An incisional biopsy of this osteolytic component was carried out on the left side of the lesion and the specimen was referred to histopathological examination. The hematoxylin and eosin (H&E) stained sections showed a combined epithelial and mesenchymal lesion. The first component revealed an odontogenic epithelial lesion predominantly cystic, but with solid areas, exhibiting epithelium with palisaded basal cells with hyperchromatic nuclei, focal reverse polarization and upper layers with stellate-reticulum-like cells (Fig. 2a,c). The mesenchymal component revealed a lesion with high cellularity, with the predominance of ovoid fibroblasts and disorganized collagen fibers permeated by numerous multinucleated giant cells of different sizes (Fig. 2b,c). To exclude brown tumor of hyperparathyroidism (BTH), laboratory exams were run and showed normal serum levels of calcium, alkaline phosphatase, and parathormones. Then, the diagnosis of ameloblastoma associated to CGCL was confirmed.

After that, it was performed marsupialization on cystic component in order to promote bone formation decreasing the cystic cavity. After three months, the whole lesion was removed by enucleation with peripheral osteotomy. Once again, the specimen was submitted to microscopic examination and the diagnosis of UA associated to CGCL was confirmed. Immunohistochemistry against CD68 highlighted the multinucleated giant and the mesenchymal cells (Fig. 2d), whereas cytokeratin 19 (CK19) and high molecular weight cytokeratin (pan-CK) stained the odontogenic epithelium (Fig. 2e,f).

The last radiography was taken in May 2019 (Fig. 1e) and revealed decreasement of the lesion, indicating bone repair. Regular follow up has not shown recurrence after 10 months of the surgical treatment.

## Discussion

Hybrid lesions or combined lesions have been rarely described in the literature. CGCL was reported in association with central odontogenic fibroma (COF) (7), fibro-osseous lesions (1), and with odontogenic keratocyst (8). On the other hand, few cases of hybrid lesions involving ameloblastoma have been reported. Cousin (9) reported the co-occurrence of glandular odontogenic cyst associated to ameloblastoma, Gupta (2) reported the occurrence of odontogenic keratocyst with unicystic ameloblastoma and Fregnani (10) described the simultaneous occurrence of ameloblastoma and orthokeratinized cyst.

Here we presented a case of CGCL associated with UA. Kawakami (11) previously described the presence of giant cells adjacent to a solid ameloblastoma as a reactive process, however in the current case other components of CGCL was seen, as abundance of ovoid to spindle mesenchymal cells and hemosiderin deposition. Furthermore, the origin of both lesions was investigated through immunohistochemistry, and the ameloblastic epithelium was positive for CK19, as reported by Upadhyaya *et al.* (7) and the CGCL component (mesenchymal and giant cells) was positive for CD68, as demonstrated by Liu *et al.* (12). However, the pathogenesis of those lesions remains unknown.

In cases of COF associated with CGCL three majors pathogenesis hypothesis had been pointed: a) a collision tumor, which results from the synchronous occurrence of the lesions; b) COF is the primary lesion and induces a GCGL reaction in response to trauma or other stimulus; or (c) CGCL produces growth factors, chemokines and cytokines that stimulates the proliferation of odontogenic cells, resulting on the COF (7).

Since our case is the first one described in the literature, there are no previous studies about its pathogenesis. Based on theories described above, clinical history and radiographic examinations, this case seems to be a real collision tumor for two majors reasons: first, the primary incisional biopsy revealed just the CGCL, without signs of odontogenic lesion, leading to the conclusion that CGCL was the predominant lesion in the beginning. The second point is the fact that the patient responded well to the treatment with intralesional corticosteroids, a specific treatment against CGCL, for 18 months. Therefore the patient probably had two separated lesions that collided when the UA growed up and reached the CGCL. In addition, we forwent the theory that the CGCL would stimulate the proliferation of odontogenic cells, because if this was a typical phenomenon, we believe that the presence of odontogenic tumors associated with CGCL would be commonly seen.

Currently, there are few cases of collision tumors published, probably because these lesions are unnoticed and/or diagnosed according to one of the prominent microscopic features. This limited number of cases lead to be difficult to predict their biologic behavior (13).

In the presented case, the initial diagnosis was CGCL. Due to lesion size and patient’s age, initially a conservative treatment was carried out. The most common intralesional drug used for CGCL in the cases reported in the literature is triamcinolone acetonide (14), but dexamethasone also is applied. Body *et al.* was the first to report the use of corticosteroids in the treatment of CGCL. In their case it was administered systemic dexamethasone, which leads to lesion reduction but also to systemic complications (15). In 1994, Terry and Jacoway first reported the treatment of CGCL with intralesional corticosteroid injections and recently a meta-analytic study demonstrated a good response of aggressive and non-aggressive CGCL with this therapeutic modality (4).

Due to the good results previously described with the use of dexamethasone, this modality of treatment was applied in the current patient. With the last biopsy revealing the presence of a UA component, surgical approach was planned. The marsupialization was performed followed by enucleation of the residual lesion with peripheral osteotomy to prevent recurrence.

Some studies have reported a higher risk of recurrence for hybrid lesions or collision tumors with CGCL component, recommending a long-term follow up and careful management (13). After 10 months of follow-up of the current case, no sign of recurrence has been observed.

To the best of our knowledge, this is the first case of UA and CGCL occurring simultaneously reported in English literature. Despite the clinical, radiological and histopathological features, the exact timing of onset of the lesions, concomitantly or independently, remains uncertain. It is necessary further studies with long-term follow-up information to understand the pathogenesis and biologic behavior of hybrid lesions and collision tumors.
